# Roles of Procalcitonin and N-Terminal Pro-B-Type Natriuretic Peptide in Predicting Catheter-Related Bloodstream Infection in Severe Burn Injury Patients

**DOI:** 10.1155/2018/5607932

**Published:** 2018-11-22

**Authors:** Baochun Zhou, Jianjun Zhu, Ziruo Mao, Lijun Liu

**Affiliations:** Department of Emergency and Critical Care Medicine, The Second Affiliated Hospital of Soochow University, Suzhou 215004, China

## Abstract

**Objective:**

To investigate the characteristics of early catheter-related bloodstream infection (CRBSI) in severe burn injury patients induced by a massive aluminum dust explosion.

**Methods:**

Sixty-eight severe burn injury patients experienced a massive dust explosion in Kunshan were included in this study. Patients received central venous catheter placement, arterial catheterization to monitor blood pressure and PiCCO cardiac monitoring, tracheostomy, mechanical ventilation, analgesics and sedation treatment, and fluid resuscitation. Clinical data including age, gender, burn surface area, fluid intake and output, urine temperature, and APACHE II score information were collected from each patient. Ultrasound screening was performed to exclude heart failure, which may lead to the change of NT-proBNP. When CRBSI was suspected, 10 ml central venous blood and peripheral arterial blood were sent for testing. For patients with suspected CRBSI, the level of PCT and NT-proBNP were monitored every day until the infection was controlled.

**Results:**

Among the 68 patients, 29 showed CRBSI. The most common pathogenic bacteria of CRBSI were *A. baumannii* (39.8%), *P. aeruginosa* (26.4%), and *K. pneumoniae* (13.7%). Procalcitonin (PCT) (2.98 ng/ml) and NT-proBNP (355 pg/ml) were significantly associated with CRBSI results. The sensitivity of PCT, NT-proBNP, WBC, and CRP was 94.2%, 89.7%, 88.3%, and 90.5%, respectively (*P* < 0.05). The area under curve (AUC) of PCT combined with NT-proBNP for prediction of CRBSI was 0.981, and the sensitivity and specificity was 0.812 and 0.857, respectively.

**Conclusion:**

PCT and NT-proBNP combination improves the diagnosis of CRBSI. PCT and NT-proBNP may be alternative candidates for potential prediction of CRBSI in patients with severe injury.

## 1. Backgrounds

Catheter-related blood stream infection (CRBSI), one of the most frequent and lethal nosocomial infections, is an important cause for hospital-acquired infection with a high morbidity and mortality. Individuals with extensive skin injury and rupture of anatomic barrier are reported to show high risks of CRBSI. Generally, the symptoms of CRBSI are usually vague as they are covered by systemic signs and sustained inflammatory reaction resulting from severe thermal injury. In clinical setting, delayed diagnosis is still the major challenge due to the lack of diagnostic markers with high sensitivity and specificity [1–4]. Therefore, it is necessary to identify potential biomarkers of CRBSI in a population with severe burn injury.

Procalcitonin (PCT) has been considered as a serum marker for assessing infection and systemic inflammatory response syndrome (SIRS) [5]. Recently, it has been used to evaluate infection in patients with burn injury [6, 7]. However, in patients with severe blast injury, little is known about the accuracy of PCT in CRBSI diagnosis [8–10]. Brain natriuretic peptide (BNP) and N-terminal pro-B-type natriuretic peptide (NT-proBNP), two important markers of heart failure recently reported to be associated with infection, are also important predictors of infection-related mortality in nonburn sepsis patients [11]. However, rare studies have been carried out to investigate the roles of these markers in predicting CRBSI [12].

In this study, serum PCT and NT-proBNP in patients with severe burn were detected in a dynamical manner. We aim to evaluate the potential roles of serum PCT and NT-proBNP in predicting CRBSI in patients with severe explosion injury.

## 2. Material and Methods

### 2.1. Patients

This is an observational study of a cohort of patients with burn injury in August 2014 in Kunshan. Patients admitted to the ICUs in the Second Affiliated Hospital of Soochow University, the First Affiliated Hospital of Soochow University, Suzhou Municipal Hospital, and the First People's Hospital of Kunshan were included in this study. The end point of this study was mortality or transfer of patients on day 90 after ICU hospitalization. Severe burn was formulated in accordance with the Chinese consensus of severe burns in 1970 in which total burn is categorized as a surface area with more than 50% burn [13]. Patients with a total burn area of less than 50% were not included in this study. Written informed consent was obtained from the relatives of the patients. The study protocols were approved by the local ethics committee.

### 2.2. Clinical Treatment

Upon ICU admission, all patients received general management including central venous catheter placement (two-lumen central venous catheterization, Arrow), Foley catheter placement, fentanyl and midazolam for analgesia and sedation, tracheal cannula or tracheostomy, mechanical ventilation, invasive arterial catheter placement to monitor blood pressure, continuous cardiac output monitoring, fluid resuscitation, and target temperature management (target temperature: 36-37°C). Besides, wound care including partially exposed or exposed care was given to these patients. For circumferential burns (degree three) or tension scar, escharotomy was performed to prevent ischemia and necrosis of the distal or deep tissue or to correct restrictive respiratory or circulatory function. Broad-spectrum antibiotics (Carbapenems) were used to prevent infection. After the acute phase, all patients continuously received mechanical ventilation, analgesics and sedation, nutritional support, debridement, and skin grafting.

### 2.3. Diagnosis of CRBSI

The diagnostic criteria of CRBSI were in accordance with the guidelines of the Infectious Disease Society of America [14]. Upon suspicious symptoms of CRBSI including fever, chills, hypotension, and SIRS, 10 ml central venous blood and peripheral arterial blood were sent for aerobic or anaerobic culture using BACTEC-9120, VITEK-32 automatic microbial analysis system and supporting culture bottles, bacterial identification card, and anaerobic culture kits and medium (bioMérieux, France). The culture temperature was set at 35.5°C. Negative report timeline was usually set as 5 days after incubation. When positive results were reported, the cultures were implanted to the corresponding medium and the pathogenic strains were isolated by aerobic and/or anaerobic culture at 36°C. All the isolated strains obtained from the anaerobic culture were subject to oxygen tolerance test.

### 2.4. Data Measurement

For each patient, the circumstances of insertion and the parameters related to indwelling catheters were recorded including fluid infusion quantity, antibiotic utilization, indwelling days, maintenance frequency and material, fever, and blood culture. Sampling of each removed catheter was conducted to monitor catheter-related infections. Strict aseptic procedures were carried out to avoid contaminating the wound near the insertion site. Ultrasound screening was performed, and the cardiac parameter (LA diameter, LA diameter, LV systolic dimension, ventricular septal thickness, and ventricular septal thickness) were recorded every 3 days. For patients with suspected infection, the level of PCT and NT-proBNP (Roche Diagnostics) were monitored every day until infection was controlled.

### 2.5. Statistical Analysis

Data with normal distribution were expressed as mean ± standard deviation (SD). These data were analyzed with SPSS 19 software. The specificity and sensitivity of PCT, NT-proBNP, WBC, and CRP for CRBSI were evaluated, and ROC (receiver operating characteristic) curves were plotted. *P* < 0.05 was considered to be statistically significant.

## 3. Results

### 3.1. Patients' Characteristics

A total of 68 patients (male: 41; female: 27; mean age: 38.3 ± 7.3 years) were finally included in this study (Table 1). The burn area ranged from 75% to 99%. The mean duration of ICU stay was 71.2 ± 17.8 days. APACHE II score at admission was 22.3 ± 7.6. About 90 days after admission, 21 patients died, 25 patients received further treatment in the ICU for unhealed large wound or respiratory support, and 22 patients were transferred to the regular wards.

### 3.2. Bacterial Spectrum of CRBSI Catheters

**Figure 1:**
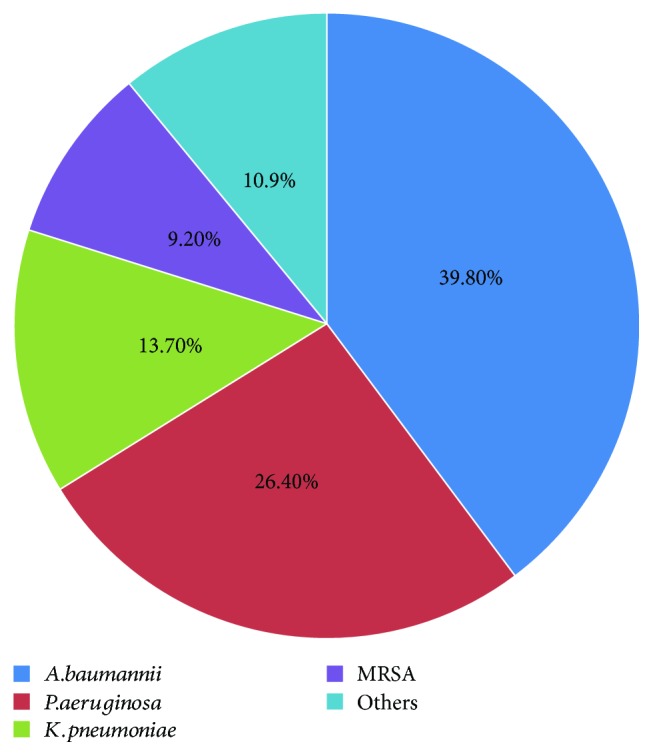
Bacterial spectrum of CRBSI-positive catheters.

Bacterial spectrum of CRBSI-positive catheters is shown in Figure 1. The most common pathogenic bacteria were *Acinetobacter baumannii* (39.8%), followed by *Pseudomonas aeruginosa* (26.4%), *Klebsiella pneumoniae* (13.7%), and *Methicillin*-resistant *Staphylococcus aureus* (MRSA, 9.2%).

### 3.3. Body Temperature, WBC Count, CRP, PCT, NT-proBNP, and Fluid Balance during the First Week in the ICU

On the first day after burn, the average body temperature decreased. Thereafter, the average temperature increased steadily from day 2 to day 7 (Table 2). The average WBC count was significantly elevated on day 1 after burn and then declined back to normal by day 4. CRP had a slight increase but no obvious trend in changing. PCT was significantly elevated on day 1 after burn and then decreased each subsequent day. On day 7, PCT was still higher than normal. No significant correlation was noticed between NT-proBNP and fluid balance (*P* > 0.05).

### 3.4. Echocardiogram of Patients Every 3 Days during Hospitalization

Table 3 shows the echocardiogram of the patients performed every 3 days during hospital stay. Our data showed that there were no obvious abnormalities in cardiac function among these patients (Table 3).

### 3.5. ROC Curve Analysis

ROC curves were depicted to evaluate the specificity, sensitivity, and accuracy of PCT, NT-proBNP, WBC, and CRP in CRBSI within 2 weeks. The sensitivity of PCT and NT-proBNP were 90.9% and 100% during the first week, respectively (Table 4). This indicated that PCT and NT-proBNP were statistically significant to predict CRBSI during the first week. However, CRP and WBC were not found to be sensitive during this period (*P* > 0.05). Additionally, during the second week, the sensitivity of PCT, NT-proBNP, WBC, and CRP was 94.2%, 89.7%, 88.3%, and 90.5%, respectively (*P* < 0.05, Table 5). This implied that PCT, NT-proBNP, WBC, and CRP were markedly sensitive for predicting CRBSI (*P* < 0.05). The area under curve (AUC) of PCT combined with NT-proBNP for prediction of CRBSI was 0.981, and the sensitivity and specificity were 0.812 and 0.857, respectively.

## 4. Discussion

Indwelling central venous catheterization is necessary for the management of critically ill patients. This increases the risk of CRBSI. Infection is the main cause of death in well-managed patients with severe burn injury [15]. Therefore, prediction of CRBSI is more important in clinical implications. In the present study, patients with severe burn injury in Kunshan were almost at a young age with a healthy condition before injury. We found that the leading three bacterial species were *A. baumannii*, *P. aeruginosa*, and *K. pneumonia*. Our data showed that PCT and NT-proBNP combination improves the diagnosis of CRBSI. PCT and NT-proBNP could serve as candidates for predicting CRBSI.

In a previous study, gram-negative bacteria are the major pathogenic bacteria in burn patients [15], which differ from the pathogenic bacteria of CRBSI in nonburn ICU patients [16]. The elevation of WBC, NT-proBNP, and CRP caused by excessive inflammatory reactions in burn patients may affect their efficiency for prediction of infection [17–19]. WBC and N-proBNP could be easily influenced by many factors, such as severe stress, trauma, bleeding, and certain medications (e.g., steroids). CRP is an acute phase reaction protein synthesized by the liver and mediated by interleukin-6 (IL-6) and tumor necrosis factor (TNF) [20]. Prior studies indicated that CRP could not be used as a marker of infection in burn patients [4, 21]. In this study, WBC and CRP could not be utilized to predict CRBSI in the first week, but they were markedly associated with CRBSI during the second week.

PCT, a precursor of calcitonin, is produced by the liver, fat cells, lung, and muscle cells [22]. Lipopolysaccharide (LPS) and sepsis-related inflammatory factors such as TNF-*α* and IL-6 can regulate the secretion of PCT [23]. Severe inflammatory factors, including IL-6 and TNF-α that exist in severe burn patients, contribute to the secretion of PCT [22]. Indeed, it may be a mechanism in which PCT showed initial increase. PCT has been considered as a marker of infection [5], but there are substantial disputes on its ability in predicting infection in patients with burn injury [1, 6, 7, 24, 25]. Partly, such uncertainty may result from a wide range of infection including wound, pulmonary, urinary tract, and blood, which may elicit differing PCT responses. We are aware that there is no specific study on PCT for the prediction of CRBSI in burn patients [8–10]. Our study indicated that PCT might serve as a candidate biomarker for predicting CRBSI in burn patients.

BNP and NT-proBNP are neurohormones secreted from cardiac ventricles in response to left ventricular strain or fluid overload [26]. At present, BNP is mainly used as a biomarker for heart failure [27]. Among these young and adult cases, cardiomyopathy indicated no obvious changes in the cardiac function. Therefore, no correlation was noticed between NT-proBNP elevation and cardiac function. Recently, several studies have reported that BNP and NT-proBNP are also elevated in the setting of infection [28–31]. In a previous study, the severity of the infection was positively associated with the elevation of BNP [32]. Our data indicated that NT-proBNP was highly sensitive and reasonably specific for predicting CRBSI in burn patients, which was superior to PCT. Under normal conditions, NT-proBNP is a nonactive precursor of BNP cleared primarily by the kidney [33]. In case of infection, the clearance of BNP is impaired [34], which may trigger an elevation of NT-proBNP in CRBSI. Besides, further studies are required to investigate the potential mechanisms. The combination of PCT and NT-proBNP contributed to the diagnosis accuracy of CRBSI, which was in line with the previous study [35].

Indeed, there are some limitations in this study. The patients included in this study were those who suffered from burn injury due to sudden onset of explosion that was uncontrolled. Besides, this study was not a randomized control study.

In summary, PCT and NT-proBNP combination improves the diagnosis of CRBSI. PCT and NT-proBNP may be alternative candidates for the potential prediction of CRBSI in patients with severe injury.

## Figures and Tables

**Table 1:** General information of the patients.

General information	Value
Gender (male/female)	47/21
Age	38.3 ± 7.3
Total burn surface area (%)	91.5 ± 7.1
Burn surface area of stage II partial thickness (%)	23.6 ± 4.5
Burn surface area of stage II full thickness (%)	50.1 ± 5.7
Burn surface area of stage III (%)	21.7 ± 8.3
ICU length of stay (days)	71.2 ± 17.8
Initial time of CRBSI (ICU day)	10.6 ± 3.4
Admission APACHE II score	22.3 ± 7.6
Outcome (90 days)	
Death	21
Continuous ICU management	25
Transferred to regular wards	22

**Table 2:** Body temperature, white blood cell, CRP, PCT, and fluid balance during the first week in the ICU.

Data	D1	D2	D3	D4	D5	D6	D7
Temperature (°C)	35.19 ± 1.02	36.22 ± 1.55	36.44 ± 0.92	36.52 ± 0.90	36.74 ± 1.40	36.96 ± 0.94	37.39 ± 1.04
WBC (10^9^/l)	36.29 ± 13.91	20.16 ± 5.20	10.80 ± 3.52	8.03 ± 2.41	7.49 ± 3.17	8.93 ± 4.27	8.41 ± 2.77
CRP (mg/l)	37.26 ± 24.29	72.77 ± 34.97	70.53 ± 38.85	67.42 ± 44.82	62.79 ± 31.35	90.01 ± 40.63	96.53 ± 49.91
PCT (ng/ml)	11.83 ± 8.11	7.78 ± 5.20	4.89 ± 3.92	2.72 ± 1.88	1.62 ± 1.22	1.57 ± 0.94	1.34 ± 0.90
NT-proBNP (pg/ml)	100 ± 22	160 ± 30	200 ± 11	250 ± 41	180 ± 33	120 ± 29	310 ± 51
Fluid balance (ml)	4791 ± 324	1782 ± 418	2928 ± 2356	2297 ± 504	4289 ± 482	3290 ± 120	3324 ± 107

D1–D7: day 1 to day 7.

**Table 3:** Cardiac parameter followed by echocardiogram.

Cardiac parameter	Minimum	Maximum	Mean ± SD
LA diameter (mm)	30.7	46.6	37.9 ± 4.4
LV diastolic dimension (mm)	41.0	54.2	49.6 ± 3.4
LV systolic dimension (mm)	26.5	39.8	31.7 ± 3.9
Ventricular septal thickness (mm)	8.1	12.0	9.4 ± 3.1
LV ejection fraction (%)	58.0	74.0	66.1 ± 5.2

**Table 4:** ROC curve analysis of PCT, NT-proBNP, WBC, and CRP during the first week.

Parameter	Area under the curve	Best cutoff value	Sensitivity	Specificity	Youden index	95% CI	*P* value
PCT (ng/ml)	0.856	2.98	0.909	0.583	0.492	0.743~0.970	0.000
NT-proBNP (pg/ml)	0.963	355	1.000	0.625	0.625	0.918~1.00	0.000
WBC	0.514	12	0.682	0.583	0.265	0.342~0.687	0.088
CRP (mg/ml)	0.190	55	0.545	0.833	0.378	0.062~0.319	0.065

**Table 5:** ROC curve analysis of PCT, NT-proBNP, WBC, and CRP during the second week.

Parameter	Area under the curve	Best cutoff value	Sensitivity	Specificity	Youden index	95% CI	*P* value
PCT	0.793	3.67	0.942	0.523	0.502	0.861~0.967	0.000
NT-proBNP	0.849	285	0.897	0.661	0.629	0.907~0.994	0.003
WBC	0.722	19	0.883	0.514	0.556	0.782~0.938	0.014
CRP	0.698	72	0.905	0.679	0.611	0.707~0.929	0.009
PCT + NT-proBNP	0.981		0.812	0.857	0.753	0.853~0.908	0.000

## Data Availability

All the data were available upon appropriate request.
